# The prevalence and correlates of falls in adults with attention-deficit/hyperactivity disorder: cross-sectional study

**DOI:** 10.3389/fpsyt.2025.1673400

**Published:** 2025-10-08

**Authors:** Maraheb M. Alkhalidi, Donald H. Lein, Mansour M. Alotaibi

**Affiliations:** ^1^ Department of Community Medicine and Behavioral Science, Faculty of Medicine, Kuwait University, Kuwait City, Kuwait; ^2^ Department of Physical Therapy, School of Health Profession, The University of Alabama at Birmingham, Birmingham, AL, United States; ^3^ Department of Rehabilitation, Faculty of Applied Medical Sciences, Northern Border University, Arar, Saudi Arabia; ^4^ Center for Health Research, Northern Border University, Arar, Saudi Arabia

**Keywords:** attention-deficit/hyperactivity disorder (ADHD), psychostimulant medication, falls, adults, prevalence, cross-sectional

## Abstract

**Background:**

Falls are a major concern due to potentially causing injuries and long-term disabilities. Studies have shown that individuals with Attention-Deficit/Hyperactivity Disorder (ADHD) have decreased balance. Poor balance is associated with increased risk of falls. Limited studies have investigated the risk of falls among adults with ADHD despite the increasing prevalence of ADHD world-wide.

**Objective:**

This study aimed to estimate the prevalence of falls and fall-related injuries, as well as to explore correlates of falls among adults with ADHD during off- and on-medication status.

**Methods:**

Adults with ADHD were recruited for this cross-sectional analysis. Participants completed two sessions (an off-medication and an on-medication session). A customized questionnaire was used to collect relevant data, including demographic information, psychostimulant medication use, fall history, and fall risk. Further, ADHD symptoms were assessed using the Adult ADHD Self-Report Scale-5. Participants completed body mass (kg) and body height (cm) measure on the first session. Participants also completed several measures of physical function, including maximum voluntary contraction (MVC [N.m]) and passive range of motion (ROM [°]) static balance assessment on a force platform (sway area [cm^2^] and sway velocity [cm/s]), Timed Up and Go test (TUG[s]) and the Lateral Step-Up Test (LSUT; [repetitions]). Prevalence of falls was estimated using frequency data. Associations of fall factors among adults with ADHD were evaluated using chi-square tests and Spearman’s correlation.

**Results:**

Forty-five adults (35 women; mean age = 28.4 ± 6.3 years) were enrolled in the study. The 12-month prevalence of falls among adults with ADHD was 37.8%. Fallers were significantly more likely to feel unsteady (62.5%), and 77.8% expressed worry about falling compared to non-fallers. The higher prevalence of falls observed among adults with the combined subtype of ADHD (46.7%). The SLEC score, PROM, TUG, and PMVC demonstrated insignificant correlation with falling in adults with ADHD during off- and on-medication status (*r_s_* = <0.25, *p* > 0.05); thus, regressions analysis for these potential correlates were not performed.

**Conclusions:**

Adults with ADHD exhibited increased fall rates. Biological sex, ADHD subtype, perceived feeling of unsteadiness, and worries about falling were associated with falls in this population.

## Introduction

Attention-deficit/hyperactivity disorder (ADHD) is a common neurodevelopmental disorder that can adversely affects motor function in children and adults (1–4). ADHD prevalence is estimated at 7 million US children aged 3–17 years in 2022 (5). The prevalence of ADHD among adults is estimated to be 6.7%, of which approximately 2.6% of the individuals have persisted ADHD since childhood (6). The Diagnostic and Statistical Manual of Mental Disorders, fifth edition (DSM-5) categorize ADHD into three subtypes: predominately inattentive, predominately hyperactive-impulsive, or combined (7). ADHD is more prevalent in men than women with a ratio of 3:1 (8, 9). In addition, ADHD is commonly associated with impairment in functional domains (self-care, mobility, cognition), and instrumental functioning activities (days out of role, productive role, and social role) when measured by WHO Disability Assessment Schedule (10, 11). These impairments may contribute to significant challenges related to maintaining attention to the surrounding environment and therefore increasing the risk of physical injuries (12).

ADHD symptoms may lead to significant physical injuries, resulting from accidents, traumas, and falls throughout adulthood (13). A systematic review and meta-analysis found that children, adolescents, and adults with ADHD were two times more susceptible to unintentional injuries compared to controls (Pooled *OR*:2.1) (14). Specifically, adults with ADHD have higher rates of falls within a one-year period in comparison to adults without ADHD (27 falls vs. 0 falls) (15). This phenomenon has been linked to significant balance impairments in adults with ADHD (15–17). Yet, there is limited evidence on the prevalence of falls and fall-related injuries among this population, and how these vary across the ADHD subtypes. This gap highlights an important issue because falls are associated with significant negative impacts such as loss of independence, skeletal fractures, and increased morbidity and mortality rates (18, 19). Psychostimulant medications, such as methylphenidate (MPH) and amphetamine(AMP) might enhance balance and motor control in adults with ADHD (16, 20–22) in addition to their roles in improving attention and executive function (23, 24). Thus, psychostimulants may potentially reduce the risk of falls in this population. This claim is supported by a population-based cohort study that found that these medications may have a protective effects against unintentionally injuries, including falls (25).

Given the abovementioned research literature gaps, our study aimed to estimate the prevalence of self-reported falls and fall-related injuries among adults with ADHD. A secondary aim was to explore the correlates of the self-reported falls. The hypothesis was that there will be an increased prevalence of falls in adults with ADHD (~33% or greater). Evidence repeatedly showed that balance performance (26, 27) and perceived risk of falls (28) are key factors in fall rates in middle-aged and older adults. Thus, the secondary hypothesis was that TUG score, SLEC score (s), and fall risk score will be correlates of self-reported falls in the same sample across off- and on-medication status.

## Methods

### Study setting, design, and participants

The cross-sectional analysis of baseline data collected from a previous study. While the parent study was designed to investigate the effect of psychostimulants on static balance in adults with ADHD (21). The current analysis focused on baseline data to estimate the prevalence of falls among adults with ADHD and to examine their associations with demographic, clinical, and behavioral characteristics. Recruitment strategies included the distribution of flyers across campus, advertisements in the University of Alabama at Birmingham (UAB) Reporter (https://www.uab.edu/reporter/), and sending email invitations to participants using data from hospital records. Data collection commenced in May 2021 and ceased in February 2022. To be enrolled in this study, participants met the following inclusion criteria: a) aged 20–55 years, b) diagnosed with ADHD by a physician or a psychologist, c) prescribed with MPH or AMP-based PS to control ADHD symptoms for a minimum of three months, d) free of major orthopedic, cardiovascular, neurological, or respiratory diseases, e) proficient in English speaking and reading, and f) ambulating freely in the community. Participants were excluded if they reported the use of assistive devices for mobility, difficulties following instructions, and the use of medications that affect movement regulation and neural firing excitability, such as anticonvulsants The age range was limited in this study to minimize the confounding effects of age-related comorbidities on fall risk. Ethical approval for the current study was obtained from the UAB Institutional Review Board under protocol number: IRB-300006200. Participants provided written informed consent prior to their enrollment in the study.

### Study procedures

**Figure 1:**
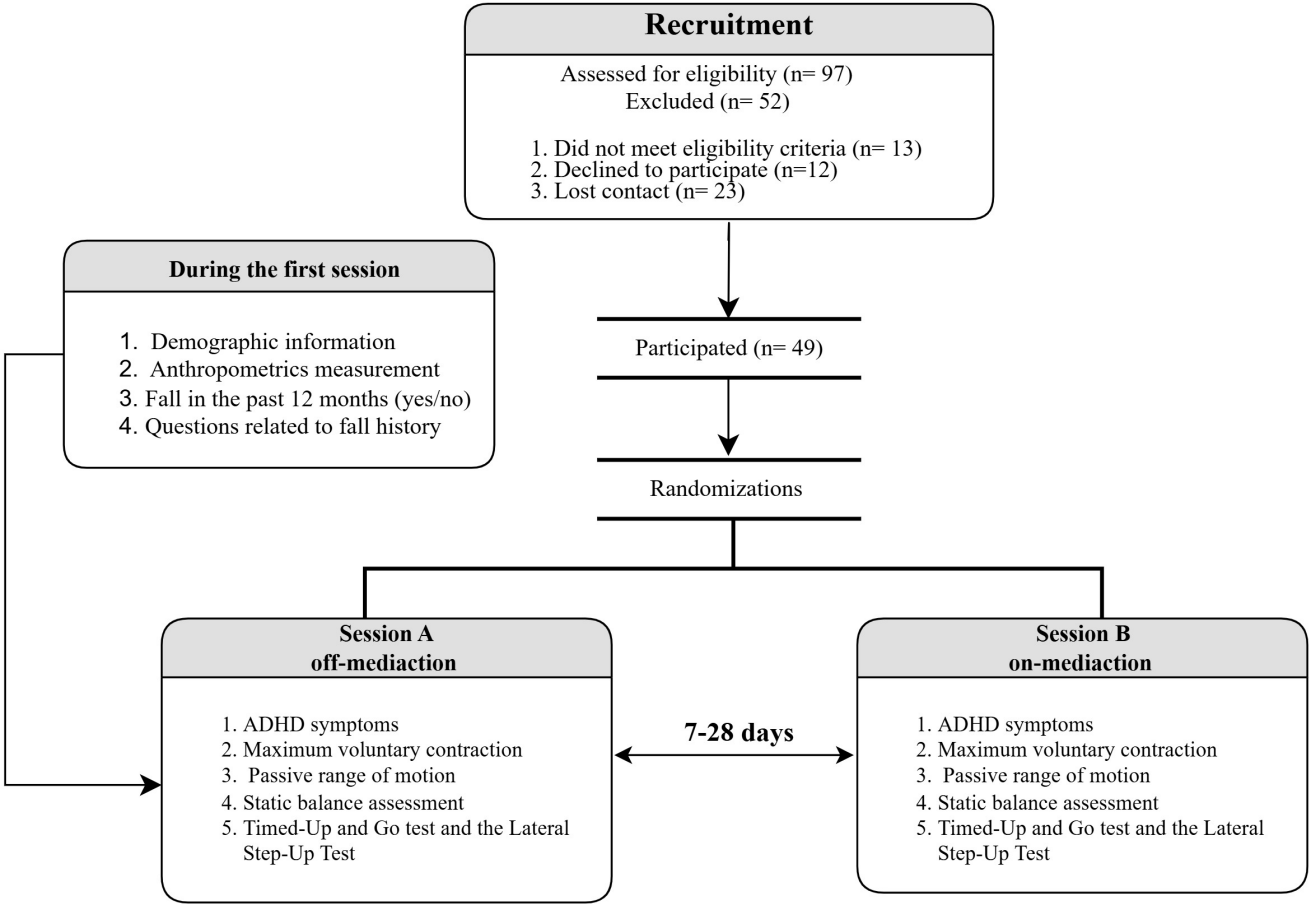
Flowchart of the study procedure.

Participants completed testing in two separate sessions: an off-medication session and an on-medication session, with an interval of 7–28 days scheduled between each of the two sessions. For the off-medication session, participants were instructed to skip their PS medication 24 h before the session to minimize the systematic effects of PS during the tests. For the on-medication session, participants were asked to consume their medication as prescribed before the session. Regardless of medication status, during the first session, participants completed a customized questionnaire that have questions about demographics, psychostimulant medication use, fall history, and fall risk assessment. Their body mass and body height measures were also assessed using a weight scale and a stadiometer, respectively. In both sessions, participants completed the Adult ADHD Self-Report Scale for DSM-5 (ASRS-5), finished maximum voluntary contraction (MVC) and passive range of motion (ROM) tests of the non-dominant leg, and performed static balance tests. Participants also completed the Timed-Up and Go and the Lateral Step-Up tests (**Figure 1**).

### Outcome measures

#### Fall risk assessment and history questionnaires and tests

The fall assessment started with three questions that asked a participant if they: 1) have fallen in the past year, 2) feel unsteady when standing or walking, and 3) worries about falling (29). If a participant reported falling in the past year, further questions were added to the previous questions, including the perceived cause of fall, fall direction (i.e., forward, backward, or sideways), location of fall (indoor vs outdoor), and whether the fall was associated with injury. If an injury resulted from a fall, more information about injury classification (e.g., wound, or fracture) and location(s) of injury(ies) were collected. In addition, a 12-item fall risk assessment questionnaire followed the previous questions about falls to understand perceived risk of falling (**Supplementary Table 1**). The Scoring of fall risk was based on the number of the questions that were answered with “yes” or “no” (30). A score of 4 or greater indicates a high risk of falls. The fall risk assessment questionnaire is highly valid and reliable for assessing fall risk in older adults (31). Further, a medication review was performed to determine the risk of falling, specifically for antidepressants and antipsychotics (e.g., benzodiazepines). The TUG test was further conducted to assess the risk of falls. Participants practiced the tests before data collection. As a part of the test, participants were instructed to stand up from a 45-cm height armchair, walk at their typical pace around a cone placed three meters from the chair, then walked back to the chair and sat down. Using stopwatches, we recorded the time (s) elapsed to complete the task. Participants performed three trials of the test, with 60-s rest intervals between the trials, and the average of these trials was recorded. If participants needed ≥12 seconds for completing this test, they were identified as at high risk of fall (29). TUG has excellent test-retest reliability in healthy adults (ICC > 0.97) (32) and a valid test for dynamic balance in adults with balance impairments due to vestibular hypofunction (33). Furthermore, TUG has a specificity of 74% and a sensitivity of 31% in adults (34). Although its predictive accuracy ability for falls is limited, TUG remains the common clinical test to assess functional mobility and balance as a quick practical assessment.

### Demographic and anthropometric characteristics

A customized self-reported questionnaire was designed to collect information on sociodemographic and clinical data, including age, sex, race, ADHD medication information, and education level. We also measured body mass (kg) and body height (cm) using a weight scale (Garmin Ltd., Southampton, United Kingdom) and a stadiometer (Charder HM200P Stadiometer, Taichung City, Taiwan), respectively. Body mass index (BMI) was calculated by dividing body mass by body height squared (kg/m^2)^, and participants were categorized based on World Health Organization (WHO) standards as follows: underweight: <18.5; normal weight: 18.5 to <25.0; overweight: 25.0 to <30.0; or obese: ≥30 (35).

### ADHD symptoms: survey ASRS-5

An updated version of WHO Adult ADHD Self-Report Screening Scale for DSM-5 (ASRS-5) was administered to participants in order to assess symptoms of ADHD (36). The ASRS-5 questionnaire consists of 6- items with a 5-point liker-type response scale ranging from ‘never’ (0) to ‘very often’ (4) that are based on the Composite International Diagnostic Interview for DSM-5 (CIDI-5.0), designed for adults aged 18 years or older with an IQ in the normal range (IQ >=80) (36). The scale demonstrates excellent sensitivity (91.4%), specificity (96.0%), and an area under the curve (AUC) of 0.94 in detecting symptoms of ADHD (36). In addition to measuring ADHD symptomology, the ASRS-5 determines the effects of PS on ADHD symptoms, with a cut-off score (14 or greater) set per the proprietary scoring rules for the DSM-5 developed by New York University and Harvard University (36).

### Ankle dorsiflexion range of motion

Ankle dorsiflexion passive range of motion (DF-PROM) was used to assess ankle joint osteokinematic movements and to determine the ankle joint ROM in degrees. As part of the assessment, the investigator manually moved the ankle joint through its range of motion and assessed the end feel of the joint, using a goniometer. An investigator trained as a physical therapist assessed the DF-PROM of the same leg that was tested for MVC test (i.e., the non-dominant leg). Measurement of ankle DF-ROM using goniometer had excellent test-rest reliability in healthy adults (ICC = 0.96) (37).

### Isometric muscle strength: plantarflexors maximum voluntary contraction

Before testing, participants performed a 5-minute walk on a treadmill for a lower extremity warmup. To ensure a relatively light warm-up, participants were asked to provide their rate of perceived exertion (RPE) several times their warm-up and maintain a score between 6 and 11 to ensure light exertion (38). Participants then completed a plantarflexors maximum voluntary contraction (PMVC) test of the non-dominant leg for consistency using an isokinetic dynamometer Biodex System 3 ™ device (Biodex Medical Systems, Shirley, NY, USA). Participants performed the test from a sitting position with their tested knee flexed at 60°. Participants’ shoulders, lower back, pelvic, and mid-thigh were strapped to minimize the synergetic effects of other muscle groups on the test. Next, participants were instructed to push against the foot plate as hard as they could when they heard “go”. The investigator verbally encouraged participants to exert their maximum effort throughout the test during trials. Participants completed five trials of 5-seconds, with 45-seconds rest between trials. The average peak torque of ankle plantarflexors in N.m of the closest three PMVC trials was used as the score of this test. Isometric muscle strength assessment of the plantarflexors using an isokinetic dynamometer has been shown to be valid and reliable in adults (39).

### Static balance and postural sway variables

Following the PMVC assessment, Participants performed static balance tasks on a force platform (1000 Hz, AMTI, Watertown, MA, USA) in stocking feet. Participants performed four tasks for 30 seconds each: standing with their feet placed shoulder-width apart with 1) eyes open 2) eyes closed; and standing with feet-together with 3) eyes open and 4) eyes closed. Sway area (cm^2^) and sway velocity (cm/s) were calculated using a programed software with MATLAB (MathWorks Inc., Natick, MA, USA). For each trial, we excluded the first and last 5-seconds from the center of pressure (COP) trajectory analysis for accuracy (40). Further, we did not control for anthropometric variables for the static balance assessment because there were no observed correlations of postural sway area and sway velocity with body mass, body height, or BMI measures. In addition, participants performed a single-leg standing test on the non-dominant leg with their eyes closed for 30-seoncds. The time (s) elapsed before touching the ground with the other foot was recorded as the score for this test (27). These measures have been shown to be valid and reliable in assessing static balance in healthy adults without ADHD (41). Finally, in order to minimize the order effect on these tests, the order of the balance tests was randomized.

### Data processing and statistical analysis

The data were analyzed using IBM^®^ SPSS Statistics for Windows, Version 27.0 (IBM Corp., Armonk, NY, USA). The statistical significance level was accepted as α = 0.05 for all statistical tests and model building. All the variables were evaluated by the Shapiro–Wilk and Kolmogorov–Smirnov tests to assess the normality distribution. No violation of the normality assumption of each variable entered in the analyses were found. Descriptive analysis were conducted to calculate the frequencies and proportions of the categorical variables (i.e. biological sex, race, education level, ADHD subtype, and ADHD medication type) and were expressed as count (n) and percentage (%). Means and standard deviations (mean ± SD) were calculated for continuous variables (i.e. age, body height, body mass, BMI, and outcome measures (DF-PROM, PMVC, fall risk score, fall time SLEC). The Chi-square (χ2) test was used to assess whether the fall prevalence differed across categories of sociodemographic factors (i.e. biological sex, race, education level, BMI classification, and ADHD subtype). Bivariate association analyses were performed to further assess the independence relationship between variables of interest (Feels unsteady when standing or walking, worries about falling) and the outcome (fell in the past year vs. did not fall). Descriptive analysis summarized questions related to fall history (i.e., perceived cause of fall, fall direction, fall location, type of injury, part of the body injured). Hierarchical binary logistic regressions were performed to determine correlates of falls, and the primary outcome was defined as a binary variable that determined whether the participant had experienced a fall in the past 12 months (fell in the past year vs. did not fall). The correlates included SLEC score, PROM, TUG, and PMVC during off- and on-medication states. The assumptions of hierarchical binary logistic regression were evaluated. Continuous correlates should be related linearly to the logit of the outcome. Spearman’s Rho correlation analysis was conducted to assess the association between the outcome and different variables (e.g. age. biological sex, BMI, DF-PROM, SLEC score (s), PMV, TUG, and fall risk score) when off- and on-medication. The strength of correlation were considered based on Cohen’s guidelines (42, 43). Spearman’s Rho correlation coefficient (r_s_) values between 0.10 and 0.29 reflect a small correlation, values between 0.30 and 0.49 indicate a moderate correlation, and values of ≥0.5 represent a strong correlation (43). The normality assumption was checked by Shapiro–Wilk and Kolmogorov–Smirnov tests as well as visualization of the standardized residuals against their normal scores by graphing Q-Q plots. To ensure the enough power of the regression model, there were a minimum of 10 observations per correlates (44, 45). We also assessed multicollinearity using Variance Inflation Factor (VIF) and VIF values above 5 indicate multicollinearity issues (46). We tested the overall model fit by applying the Hosmer-Lemeshow goodness-of-fit test. The Spearman’s Rho correlation did not indicate considerable relationship between the outcome (fall vs no fall) and the correlates (*r_s_* < 0.25). Thus, we did not proceed with the hierarchical logistic regression and reported that SLEC score (s), PROM, TUG, and PMVC have low predictive ability of falls in adults with ADHD when off- and on-medication.

## Results

### Baseline characteristics of the study participants

Forty-five adults with ADHD completed participation in this study, of whom 35 (77.8%) were women and 10 (22.2%) were men. The predominant age range of participants was between 21.3 (5th percentile) and 44.1(95th percentile), with a mean (SD) age of 28.4 (6.3) years. Of the total study participants, 73.3% were of Caucasian (white) race, and 51.1% reported to have attained a graduate degree or higher. With regards to BMI, 42.2% of adults were classified as normal weight, and 31.1% were classified as obese (**Table 1**). The majority of participants were diagnosed with either the Inattentive subtype (n = 15; 33.3%) or Combined subtype (n = 13; 33.3%) of ADHD. The number of participants who used an AMP-based stimulant (n = 37; 82.2%) was considerably higher than those who used MPH-based stimulants (n = 8; 17.8%) (**Table 1**).

**Table 1:** Clinical characteristics of the sample study and prevalence of fall according to the participants characteristics (n = 45).

Characteristics	Total Sample	Prevalence of fall	
	% (n)	% (n)	*P*-value^*^
**Overall**	100.0(45)	37.8(17)	–
Biological Sex			
Female	77.8(35)	42.9(15)	0.189
Male	22.2(10)	20.0(2)	
Age			
Age (years), [mean(±SD)]	28.4(±6.3)		
Race			
Caucasian (white)	73.3(33)	39.4(13)	0.987
Asian	6.7(3)	33.3(1)	
Black or African American	13.3(6)	33.3(2)	
Mix of two races	6.7(3)	33.3(1)	
Anthropometrics Measures			
Body Mass (Kg) ,[mean(±SD)]	81.9(±23.9)		
Body Height (cm),[mean(±SD)]	170.5(±9.0)		
Body Mass Index (kg/m^2^),[mean(±SD)]	28.1(±7.6)		
Body Mass Index (kg/m^2^)			
Underweight (<18.5)	2.2(1)	0.0(0)	0.601
Normal (18.5 to <25.0)	42.2(19)	47.4(9)	
Overweight (25.0 to < 30.0)	24.4(11)	36.4(4)	
Obese (≥30.0)	31.1(14)	28.6(4)	
Education Level			
Did some college	17.8(8)	25.0(2)	0.695
Undergraduate	31.1(14)	42.9(6)	
Graduate Level	51.1(23)	39.1(9)	
Subtype of ADHD			
Inattentive	33.3(15)	20.0(3)	0.271
Hyperactive	2.2(1)	0.0(0)	
Combined	33.3(15)	46.7(7)	
Unspecified	2.2(1)	0.0(0)	
Not Determined	28.9(13)	53.8(7)	
Psychostimulant Medication			
MPH based (min-max dose in mg)	17.8(8) (10.0–70.0)		
AMP based (min-max dose in mg)	82.2(37) (5.0–70.0)		

* Calculated using chi-square test. *P*-value <0.05 was considered statistically significant.

### Prevalence of fall

The 12-month prevalence of falls among adults with ADHD in the total population was estimated to be 37.8% (17/45), with a higher prevalence in women than men although not statistically significantly different between the sexes (42.9% vs. 20.0%; *p* = 0.189). Furthermore, fall prevalence varied among racial groups, with highest percentage in Caucasian races, though these differences did not gain statistical significance (39.4%, *p* = 0.987). Fall prevalence were not statistically differences between the following categories of BMI: normal BMI (47.4%), overweight (36.4%) and obese (28.6%) adults with ADHD. Among ADHD subtypes, the prevalence of falls was estimated to be 46.7% in the Combined subtype. With regards to education level, adults with ADHD who held an undergraduate degree had the highest observed prevalence of falls, however, this differences was not statistically significant(42.9%; *p* = 0.695).

### Descriptive analysis of the fall history

**Table 2**. summarized the fall history in the total sample. Overall fall risk scores were higher among adults who experienced a single fall compared to those who fell twice or more (24.4%; 11 of 17 fallers). Fallers were reported less injuries but tend to have a greater percentage of falls due to perceived causes of slips (n = 7; 15.6%) followed by trips (n=4;8.9%). Approximately 22.2% of adults with ADHD reported that they experienced falls while they were in an outside setting and in the forward direction.

**Table 2:** Descriptive analysis of fall history.

Characteristics	% (n)
Overall	37.8(17)
FRQ	
Single fall	24.4(11)
Frequent fall (≥ twice)	13.3(6)
Cause of fall	
Slips	15.6(7)
Trips	8.9(4)
Sudden fall	2.2(1)
Fall direction	
Forward	22.2(10)
Backward	8.9(4)
Sideway	6.7(3)
Fall location	
Indoor	15.6(7)
Outdoor	22.2(10)
Injury associated with the fall	
Yes	26.7(12)
No	73.3(33)
Type of injury	
Skin damage (bruises)	13.3(6)
Bone injury (fracture/sprain)	13.3(6)
Part of the body injured	
Upper limb	4.4 (2)
Lower limb	20.0(20)

### Correlates of falling

Participants who reported that they fell in the past 12 months were significantly more likely to feel unsteady when standing or walking compared to non-fallers (62.5%; p = 0.011). Fallers tended to have higher rates of being worried about falling than those who did not experience it in the past 12 months (77.8%; *p* = 0.006). The results of a bivariate analysis of factors associated with falling in the past 12 months are shown in **Table 3**. Spearman’s correlation demonstrated no significant correlation between SLEC score, PROM, TUG, and PMVC and the dependent variable (fell in the past year vs. did not fall) during off- and on-medication duration (*r_s_* = <0.25, *p* > 0.05) (**Table 4**).

**Table 3:** Bivariate association measure by chi-square test.

Characteristics	Total sample	Fall in the past 12 months		
		Yes	No	*P*-value^*^
	% (n)	% (n)	% (n)	
Overall	100.0(45)	37.8(17)	62.2 (28)	–
Feels unsteady when standing or walking				
Yes	35.6(16)	62.5(10)	37.5(6)	**0.011**
No	64.4(29)	24.1(7)	75.9(22)	
Worries about falling				
Yes	20.0(9)	77.8(7)	22.2(2)	**0.006**
No	80.0(36)	27.8(10)	72.2(26)	
Biological Sex				
Female	77.8(35)	42.9(15)	57.1(20)	0.189
Male	22.2(10)	20.0(2)	80.0(8)	

*Calculated using chi-square test. *P*-value <0.05 was considered statistically significant and highlighted in bold.

**Table 4:** Spearman’s correlations.

	Descriptive	Outcome measure: fall in the past 12 months	
	Median(IQR)	Correlation coefficient (r_s_)	*P*-value^*^
Demographics			
Age(years)	27.00(7.00)	-0.19	0.217
BMI(kg/m^2^)	25.23(8.29)	0.11	0.474
On-medication			
PROM(degrees)	5.00(5.00)	0.08	0.605
SLEC (s)	7.33(17.46)	0.16	0.289
PMVC(N.m)	122.60(93.05)	0.12	0.470
TUG(s)	7.73(1.30)	0.03	0.853
Fall risk score	2.00(2.00)	-0.24	0.125
Off-medication			
PROM(degrees)	6.00(7.00)	-0.17	0.269
SLEC (s)	7.81(19.61)	0.18	0.232
PMVC(N.m)	126.50(68.45)	0.08	0.616
TUG(s)	7.78(1.28)	-0.07	0.711
Fall risk score	2.00(2.00)	-0.35	**0.017**

N = 45. BMI, Body mass index; PROM, Passive Range of Motion; SLEC(s), Fall time during Single-Leg Eyes Closed; PMVC, Peak Muscle Voluntary Contraction; TUG, Timed Up and Go. *P-value < 0.05 was considered statistically significant and highlighted in bold.

## Discussion

Our study is the first to report the prevalence of falls among adults with ADHD, with a high fall prevalence of 37.8%, exceeding rates observed in older adults aged 65 years and older (~ 30%) (47), a population known with increased risk of falls. Such findings highlight the vulnerability of this population to falls, likely driven by motor and cognitive impairments inherent to ADHD (2, 48). Sex differences in falls are presented in our findings, with women exhibiting two-fold higher fall prevalence than men, though this difference was not statistically significant. This observation aligns with prior literature suggesting that women with ADHD may experience greater motor coordination difficulties, potentially linked to hormonal fluctuations affecting attention and motor control (8, 9). Furthermore, individuals with the combined ADHD subtype exhibited the highest fall rates, consistent with findings that this subtype presents more pronounced functional impairments due to the co-occurrence of inattention and hyperactivity (49). Moreover, simple reporting of feeling unsteadiness and concern about falling were significantly related to falling in the past year, underscoring the interplay between psychological perceptions and falling. This finding aligns with studies in older adults and other neurological conditions, such as Parkinson’s disease, where fear of falling exacerbates balance impairments through inducing compensatory movements that disturb maintaining posture (33). Collectively, our findings suggest that falls in adults with ADHD may be related to psychosocial factors, such as confidence about unsteadiness and worries about falling.

Several factors may explain why individuals with ADHD may have a high prevalence of falls, including impaired executive function (48), slower reaction times (50), and impaired motor performance (2). Studies have shown that adults with ADHD demonstrate increased postural sway and instability during static balance tasks, which could increase the risk of falls compared to adults without ADHD (17, 21). Furthermore, falls in ADHD share similarities with other neurological conditions, such as multiple sclerosis, in terms of balance impairments but differ in underlying mechanisms. For instance, balance issues in multiple sclerosis are more likely to be related to demyelination and spasticity (51),whereas those in ADHD are more closely associated with attentional deficits and motor planning impairments (20, 21). Furthermore, falls have been widely investigated in dementia populations, where cognitive decline, inattention, and executive dysfunction are strongly linked to increased fall risk (52, 53). Although both ADHD and dementia involve attentional difficulties, the mechanisms are fundamentally different, with dementia reflecting age-related neurodegenerative processes, while ADHD reflects a neurodevelopmental condition persisting into adulthood (52, 53). Thus, our study adds novel evidence by focusing on ADHD-related fall risk, which has been largely overlooked compared to the more extensively studied dementia population. To these ends, further studies are needed to understand the underlying mechanisms behind the increased fall rates in ADHD and for ADHD-specific fall prevention strategies, given the unique interplay of cognitive and motor deficits.

Although we did not directly assess exercise intensity in this study, prior research have shown that engaging in regular moderate intensity exercise is associated with reduced fall risk in older adults (54). These findings highlight the potential value of future research to investigate whether structured exercise interventions might also mitigate fall risk in adults with ADHD. Regular engagement in physical activity (PA) is well-documented to enhance muscle strength, proprioception, and postural control in middle-aged adult (55), which may contribute to mitigating fall risk in diverse populations, including those with ADHD. Furthermore, an analysis of the same data of this study found a positive association between aspects of executive function and minutes/day of moderate-to-vigorous physical activity (56). In ADHD, structured PA programs, such as balance-focused yoga or resistance training, could address specific motor deficits while improving ADHD symptoms, such as hyperactivity and inattention. For example, research found that tai chi and yoga reduce fall rates by up to 45% in older adults. This is important because these forms of exercise also helped in in reducing ADHD symptoms (57). Future research may examine if these structured PA programs may hold promise in reducing the risk of falls in ADHD.

PS medications, including amphetamine- and methylphenidate-based treatments, have also demonstrated a partial protective effect on balance performance in adults with ADHD (21). Previous research supports this finding, indicating that psychostimulants enhance motor coordination and postural stability by improving dopaminergic and noradrenergic activity in brain regions that control balance (16, 58). Furthermore, medication cessation may contribute to susceptibility risk of falls due to potential side effects of medication (e.g. blurred vision or accommodation difficulties, headache, dizziness, and orthostatic hypotension) (59). Discontinuation of medication might worsen ADHD symptoms such as impulsiveness, inattention, and poor motor coordination, which may also elevate fall risk indirectly (60, 61). Collectively, combining medications with tailored physical rehabilitation and balance training may offer synergistic benefits, addressing both the cognitive and physical contributors to fall risk.

The physical injuries of falls in adults with ADHD included skeletal fractures and ligamentous sprains and were frequently reported in our sample. These injuries often reduce mobility and are associated with prolonged recovery times, all of which negatively impact quality of life (62). Although quality of life was not measured in this study, significant falls and associated injuries may have a negative impact on this factor. Evidence from older adults, a population at high-risk of falls and fall-related injuries, shows that falling and its related injuries are associated with reduced quality of life (63). Furthermore, significant injuries from falls may have an increased economic burden, including hospitalization and rehabilitation costs, which is substantial for recovery, as seen with older adults (64).

Psychological impacts may have a significant influence on the likelihood of falling. This fear can indirectly lead to activity restriction, elevating the risk of falling (65). Studies in older adults show that fear of falling is independently associated with reduced physical activity, poorer balance performance, and higher rates of depression, suggesting similar mechanisms may be at play in ADHD (13). Interventions that combine physical training with psychological support, such as cognitive-behavioral therapy (CBT), may help enhance physical function and fear of falling, thereby reducing the risk of falling (66).

Although exercise and functional tools such as the TUG test have been widely studied in fall prevention among older adults (67), our study did not evaluate exercise interventions, nor did it establish predictive relationships with falls. Given the cross-sectional design and the lack of statistically significant predictors, our results should be interpreted as exploratory rather than prescriptive. Nevertheless, our findings highlight the importance of future longitudinal and interventional research to determine predictors of falls among adults with ADHD, a group in which fall risk remains poorly characterized. Furthermore, educating healthcare providers on the high-risk of falling among adults with ADHD can raise awareness and help clinicians to take appropriate actions for reducing the risk of falls, such as referrals to rehabilitation services. From a policy perspective, integrating fall risk assessments into ADHD care protocols is essential, given the increased rate of falls. Primary care physicians may consider routine screening using tools such as fall risk questionnaires and the TUG test could help identify individuals at high risk, enabling timely interventions. Finally, emerging technologies, such as wearable devices for real-time fall detection and prevention, could also play a pivotal role in estimating the rates of falls. These devices, when paired with smartphone applications, can provide continuous monitoring and feedback, allowing both individuals and clinicians to track and manage fall risk more effectively.

### Study strengths and limitations

To the best of our knowledge, this is the first study conducted with a population sample of adults with ADHD to estimate the prevalence of falls and fall-related injuries and explore correlates of falls, which can be used to inform interventions and screening for falls within the ADHD community. Our findings can assist the health policymakers and other stakeholders in gaining a clearer understanding of the impact of ADHD, which is essential for resource planning and implementing prevention fall measurement strategies for individuals with ADHD. Furthermore, the utilization of valid and reliable clinical assessments, along with the adaptation of questions from standardized questionnaires facilitated comparisons with prior studies. The findings of this study should be interpreted with caution considering the following limitations. First, the small sample size and reliance on self-reported fall data limit its generalizability. Furthermore, the fall data was based on patient recall, which may affect results due to recall bias. In addition, the homogeneity of the sample, which was predominantly composed of female participants. These demographic characteristics may further limit the external validity of our findings to the broader adult ADHD, particularly regarding fall risk. In this study, we were primarily interested in examining if fall rates are a concern among adults with ADHD. Consequently, future studies should aim to recruit larger, more demographically diverse, populations, gender-balanced samples and incorporate objective fall-tracking/detection approaches, such as accelerometer-based devices, to minimize recall bias. Moreover, we collected cross-sectional data, hence no causal associations can be assumed. Longitudinal studies are needed to explore the temporal relationships between ADHD symptoms, physical performance, and fall risk. Investigating the combined effects of pharmacological and non-pharmacological interventions on long-term outcomes will also provide valuable insights for developing comprehensive fall prevention strategies tailored to adults with ADHD.

## Conclusions

This study highlights that falls are common in adults with ADHD. The prevalence of falls in this population is like that reported for older adults in the research literature, suggesting that falls may be a clinically relevant concern in ADHD. Nevertheless, given the lack of statistical significance of these findings, these findings should be considered exploratory. Key factors that may be related to falls included gender, ADHD subtype, subjective feelings of unsteadiness, and worry about falling, highlighting the multifactorial nature of fall risk in this group. These findings call for the integration of targeted fall prevention strategies when caring for individuals with ADHD. Furthermore, the findings from this study provides emphasis on exploring interventions such as exercise and cognitive-behavioral therapy that alleviate the fear of falling, thereby reducing the risk of falls. Future research should focus on larger, longitudinal studies to further examine the interplay of physical, cognitive, and psychological factors contributing to fall risk to help refine intervention strategies. By addressing these challenges, clinicians, policymakers, and researchers can significantly contribute to reducing the risk of falls in this population.

## Data Availability

The raw data supporting the conclusions of this article will be made available by the authors, without undue reservation.
