# Supplementary material for: The prevalence and correlates of falls in adults with attention-deficit/hyperactivity disorder: cross-sectional study
Source: Front Psychiatry. 2025 Oct 8;16:1673400. doi: 10.3389/fpsyt.2025.1673400 (PMC12540338; doi:10.3389/fpsyt.2025.1673400)
Supplement: Supplementary file 1 [file Table1.docx]

| **The Adult Self-Reported Rating Scale Screening Scale for DSM-5** | **Never** | **Rarely** | **Sometimes** | **Often** | **Very Often** |
| --- | --- | --- | --- | --- | --- |
| How often do you have difficulty concentrating on what people say to you, even when they are speaking to you directly? |  |  |  |  |  |
| How often do you leave your seat in meetings or other situations in which you are expected to remain seated? |  |  |  |  |  |
| How often do you have difficulty unwinding and relaxing when you have time to yourself? |  |  |  |  |  |
| When you are in a conversation, how often do you find yourself finishing the sentences of the people you are talking to before they can finish them themselves? |  |  |  |  |  |
| How often do you put things off until the last minute? |  |  |  |  |  |
| How often do you depend on others to keep your life in order and attend to details? |  |  |  |  |  |
| **New York University and the President and Fellows of Harvard College** |  |  |  |  |  |
| How did you sleep last night? | **☐ Poor** | **☐ Fair** | **☐ Good** | **☐Excellent** |  |
| Did you sleep your normal amount of time? | ______hours ______minutes | | | | |
|  | | | | | |

**Supplement A. Risk assessment questionnaire.**
